# HIV-1 Rev oligomerization is not obligatory in the presence of an extra basic domain

**DOI:** 10.1186/1742-4690-2-39

**Published:** 2005-06-10

**Authors:** Clemens Furnes, Thomas Arnesen, Peter Askjaer, Jørgen Kjems, Anne Marie Szilvay

**Affiliations:** 1 Department of Molecular Biology, University of Bergen, N-5020 Bergen, Norway; 2 Department of Molecular Biology, University of Aarhus, DK-8000, Aarhus C, Denmark; 3 EMBL, Heidelberg, Germany

## Abstract

**Background:**

The HIV-1 Rev regulatory protein binds as an oligomeric complex to viral RNA mediating nuclear export of incompletely spliced and non-spliced viral mRNAs encoding the viral structural proteins. However, the biological significance of the obligatory complex formation of Rev upon the viral RNA is unclear.

**Results:**

The activity of various fusion proteins based on the negative oligomerization-defect Rev mutant M4 was tested using Rev dependent reporter constructs. An artificial M4 mutant dimer and an M4 mutant containing an extra basic domain from the HTLV-I Rex protein exhibited nearly full activity when compared to wild type Rev.

**Conclusion:**

Rev dimerization appears to be required to expose free basic domains whilst the Rev oligomeric complex remains bound to viral RNA via other basic domains.

## Background

The cytoplasmic expression of unspliced and incompletely spliced HIV-1 mRNAs encoding the HIV-1 structural proteins and enzymes is dependent upon the Rev protein [1]. Rev-dependent mRNAs are characterized by two types of *cis*-acting sequences, a single Rev response element (RRE) [2,3] and several *cis*-acting repressive sequences (CRS) [4-6]. These sequences are removed in the completely spliced HIV-mRNAs, which therefore do not require Rev for cytoplasmic appearance and translation. The Rev protein, encoded by the completely spliced HIV-1 mRNA, is a nucleocytoplasmic shuttle protein that following nuclear import binds to and exports the RRE-containing RNAs to the cytoplasm [7,8]. Genetic studies of the 116 residue Rev protein have defined several functional domains; including a basic domain (aa 35–50) that specifies nuclear and nucleolar localization of Rev (NLS/NOS) in addition to specific binding of Rev to RRE [3,9-11]. An other essential domain (aa 75–84) signals active nuclear export of Rev (NES) [8,12-14]. The Rev basic domain binds with high affinity to a site within the stem-loop IIB of the RRE and also to other sites after or upon oligomerization [15]. This binding of oligomeric Rev to target RNA is important for Rev function [16]. It is, however, not clear if Rev binds as a pre-formed complex or if oligomerization occurs after binding of the first monomer to the IIB sequence. The binding of monomeric Rev to IIB may induce conformational changes in the RRE secondary structure allowing binding of additional Rev molecules stabilized by protein-protein interactions [17-19]. However, Rev oligomerization has been shown to occur independently of RRE RNA both *in vitro *[3,20-22] and *in vivo *[23-27]. The fact that Rev forms RNA-independent complexes indicates that complex formation may occur before binding to RNA. Although following binding of the first oligomeric Rev complex, additional complexes may bind to other low affinity sites within RRE. Interactions between the preformed complexes could then be mediated by residues different from those involved in the primary complex formation. This model could explain the apparently conflicting reports identifying different regions in oligomer formation. However, it is now generally agreed that sequences flanking the basic domain are involved in oligomer formation [3,20,21,23,25-27]. Of the regions reported to be essential for oligomerization, only the region N-terminal to the basic domain was found to be necessary for oligomer formation in the cytoplasm [26,28]. One of these mutants (M4) is mutated at residues 23, 25 and 26 [29]. It is not clear whether the M4 mutations directly affect the residues that are involved in the oligomer formation or if the mutations cause perturbation of the structure and thus affect the ability to form oligomers [30]. In the current study, the M4 mutant was studied to clarifying why oligomer formation is essential for Rev activity by assessing the requirements for restoration of the activity of the mutant.

## Results

### The intracellular localization of Rev and mutants

**Figure 1:**
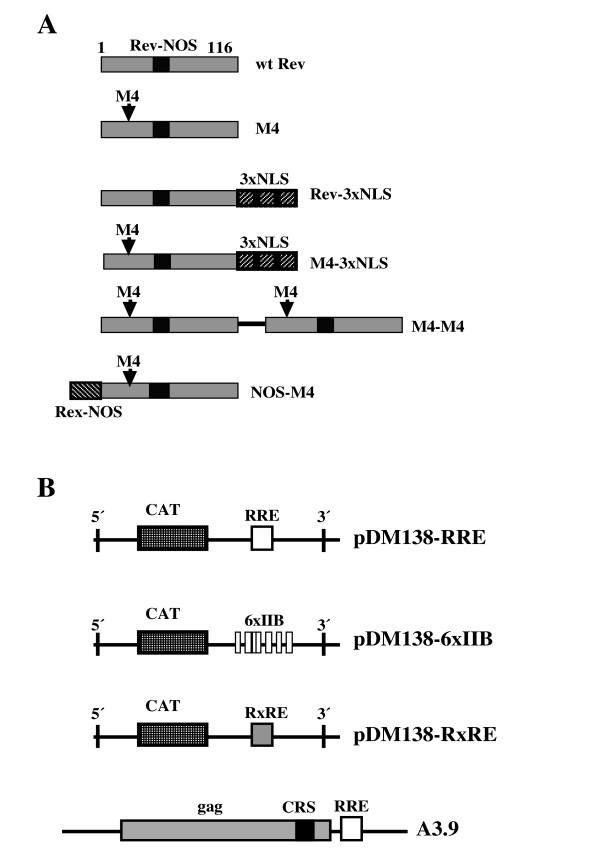
A, Schematic diagram of wild type and Rev mutants. The location of the M4 mutations are indicated by arrows. The Rev basic domain is indicated as Rev-NOS, the three copies of the large T-antigen NLS are indicated as 3xNLS, the Rex overlapping NLS/NOS signal is shown as Rex-NOS. B, Schematic diagram of the reporter systems. The CAT gene and the 5' and 3' splice sites are indicated. The Rev and Rex responsive elements are indicated as RRE and RxRE respectively. The 6 copies of the Rev high affinity binding site IIB is indicated as 6xIIB. The Rev dependent gag mRNA with the CRS element fused to the RRE expressed in the A3.9 cells is shown below. The drawings are not to scale.

**Figure 2:**
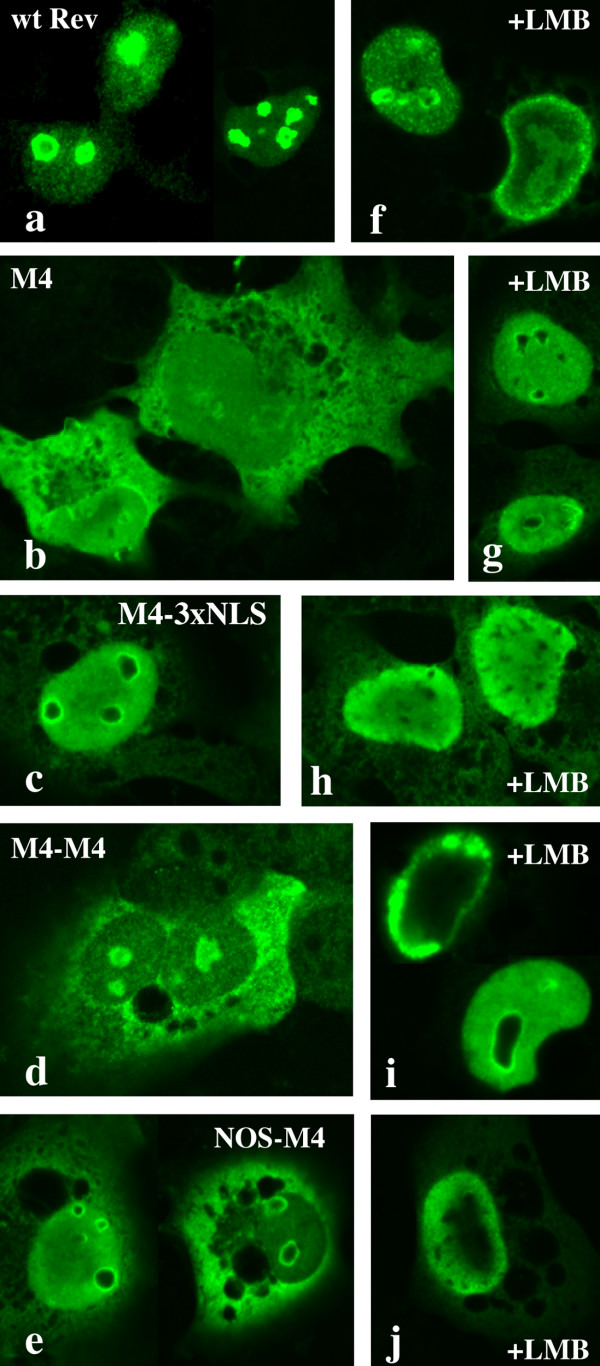
The intracellular steady state localization of the wild type Rev and mutants is shown in the panels to the left (panels a-e). The panels to the rights show the nuclear accumulation of the wild type and mutant proteins after treatment with 5 nM LMB for 6 hours (panels f-j). The anti-Rev Mab 8E7 combined with FITC labeled anti-mouse IgG2a (Southern Biotech) was used for detection of Rev and the M4 mutants.

The intracellular distribution of the M4 and the M4 derived Rev mutants (schematically outlined in figure 1) were tested by immunofluorescence in the absence or presence of 5 nM Leptomycin B (LMB) for 6 hours before fixation [31]. Wild type Rev localization was predominantly nuclear and nucleolar while the M4 mutant localized mainly to the cytoplasm with a weak nucleolar and nucleoplasmic staining (Figure 2, panels a and b). The addition of the three NLS from the large T-antigen enhanced nuclear import of the M4 mutant (Figure 2, panel c), whereas the M4-M4 dimer and the NOS-M4, which both contain two nuclear import signals, mostly localized to the cytoplasm. The nuclear staining was somewhat stronger than that of M4 (Figure 2, panels d and e). Treatment with LMB did not dramatically change the distribution of the wild type Rev protein (Figure 2, panel f). Unexpectedly, the LMB treated cells expressing the M4 mutants showed accumulation in the nucleus similarly to Rev, suggesting that the nuclear import of all the mutants occurred and that the nuclear export of the M4 mutants was mediated by an LMB-dependent pathway (Figure 2, panels g-j).

### Testing Rev activity using reporters encoding the CAT gene within an intron

**Figure 3:**
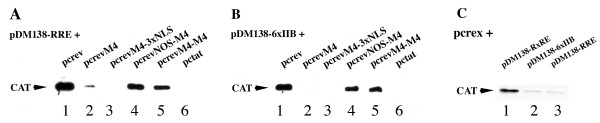
Functional analysis of wild type Rev and M4 mutants by western blot analysis of COS-7 cells co-transfected with the Rev dependent reporter plasmids pDM138-RRE (A, C), pDM138-6xIIB (B, C) or the Rex dependent reporter plasmid pDM138-RxRE (C) together with the plasmids indicated above the lanes. The CAT protein was detected by polyclonal anti-CAT antibodies (Sigma) combined with HRP-labeled anti-rabbit Ig (Amersham) and developed using ECL. The lane numbers are indicated below.

The functional activity of the mutants was tested using the two reporter plasmids pDM138-RRE and pDM138-6xIIB (Figure 1B). Figure 3 displays the results of one experiment showing the CAT expression in COS-7 cells co-tranfected with the reporter plasmids together with the M4 mutant plasmids and pc*rev*. The plasmid pc*tat *was included as a negative control. Table 1 shows the results of three or more additional and independent experiments related to the activity of wild type Rev, the negative control and to the relative amount of cell lysates in the samples. As expected, M4 displayed very low activity compared to the wild type protein (Figure 3A and 3B, Table 1). The activity of the M4-3xNLS mutant was also low using the pDM138-RRE reporter plasmid. Some of the activity was rescued using pDM138-6xIIB but addition of 3xNLS to Rev also enhanced the relative Rev activity (Table 1). In contrast to the M4 and M4-3xNLS mutants, M4-M4 and NOS-M4 were both active in co-transfection experiments using the Rev dependent pDM138 reporter plasmids containing RRE or six IIB high affinity binding sites (Figure 3A and 3B, Table 1).

**Table 1:** The relative activity of Rev and M4 mutants

	pDM138-RRE Rev activity as CAT produced (%)	pDM138-6xIIB Rev activity as CAT produced (%)
Rev (positive control)	100	100
Rev-3xNLS	81	113
M4	7	0
M4-3xNLS	10	24
NOS-M4	67	72
M4-M4	73	61
Tat (negative control)	0	0

The numbers represent the mean value of three or more independent experiments related to the activity of wild type Rev, the negative control and the amount of cellular protein.

There are conflicting reports of Rex's ability to rescue RRE RNA [32,33]. Therefore, cells were co-transfected with the Rev dependent reporters pDM138-RRE and pDM138-6xIIB together with a vector encoding Rex. The Rex dependent reporter pDM138-RxRE was included as a positive control for Rex activity. Rex dependent CAT expression was only detected when using pDM138-RxRE containing the specific Rex responsive element (Figure 3C). This indicated that the Rex-NOS sequence in NOS-M4 did not bind to IIB in the co-transfection experiments using pDM138-RRE and pDM138-6xIIB.

### Testing Rev activity using a cell line expressing HIV-1 *gag *mRNA including a CRS element

**Figure 4:**
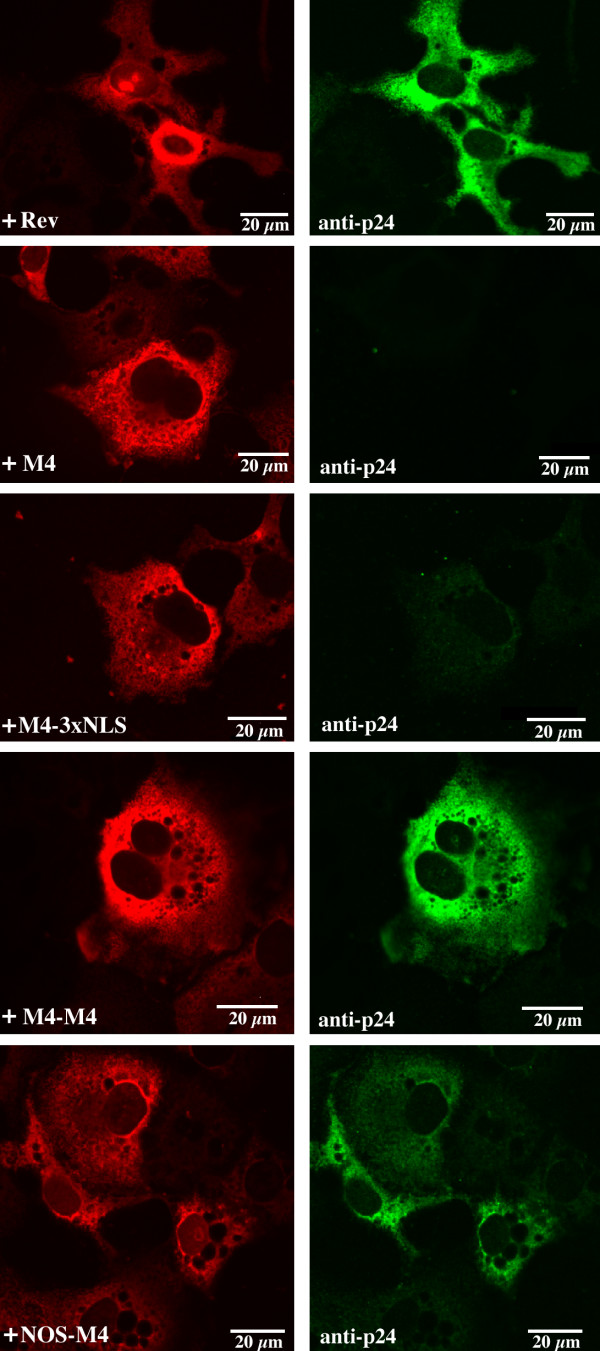
Functional analysis of wild type Rev and M4 mutants by single cell analysis of transfected methanol fixed A3.9 cells expressing Rev-dependent *gag *mRNA. The panels to the left show expression of Rev and the mutants detected by the anti-Rev Mab 8E7. The panels to the right show Gag expression in the same cells. Gag p55 was detected using the anti-p24 Mab.

Wild type Rev and the mutants were also tested by transfecting pc*rev *and the mutant plasmids into the stable cell line A3.9 expressing a *gag *mRNA fused to RRE [34]. No Gag p55 was detected in cells transfected with pc*rev*M4 and pc*rev*M4-3xNLS whilst Rev dependent Gag expression was observed in cells expressing Rev and the two mutants M4-M4 and NOS-M4 (Figure 4, right panels). However, compared to the cells transfected with pc*rev*, the number of Gag positive cells and the amount of Gag protein expressed in single cells was clearly less in cells transfected with pc*rev*M4-M4 and pc*rev*NOS-M4 (Figure 4). This trend was confirmed by western blot analysis of transfected A3.9 cells (not shown). In the A3.9 cells the cytoplasmic localization of the M4 mutants was even clearer than in the COS-7 cells (Figure 4 left panels).

## Discussion

The intracellular distribution of M4 was previously found to be mainly cytoplasmic [14,26]. Since this mutant has been shown to bind to RRE [3], an alternative explanation for the loss of function could be that cytoplasmic retention of M4 resulted in lack of M4 in the nucleus. The present study was conducted to test this hypothesis by using a combination of extra nuclear import signals for the M4 mutant and employing a reporter allowing binding of six Rev molecules to the RNA. The experiments using M4-3xNLS showed that neither efficient nuclear import nor binding of six monomers to intron RNA was sufficient for restoration of activity. Some of the activity of M4-3xNLS was rescued when the RRE sequence was replaced by 6xIIB allowing binding of six molecules to RNA. Control experiments with pc*rev*-3xNLS demonstrated that the addition of 3xNLS also enhanced the relative activity of Rev using the reporter pDM138-6xIIB (Table 1). Thus, the extra lysine rich NLS signals may have improved the nuclear import or increased non-specific binding to the viral RNA. The M4-M4 mutant comprises two NES signals whilst NOS-M4 contains only one. Both mutants were, however, highly active in co-transfection experiments using the Rev dependent pDM138 reporter plasmids suggesting that the extra NES signal in M4-M4 is not responsible for the rescue of Rev activity (Figure 3, Table 1).

There was no significant difference in activity of wild type Rev or the NOS-M4 and M4-M4 mutants whether the reporter contained RRE or six IIB high affinity binding sites. This is in agreement with previous findings suggesting that the formation of oligomeric complexes on RRE is mainly dependent on protein-protein interactions and not so dependent on the RNA sequences specificity outside the IIB site [15].

The two mutants M4-M4 and NOS-M4 also activated Gag-expression in A3.9 cells. Thus, these mutants were also active in this essentially different reporter system. The pDM138 plasmids encode mRNAs with the CAT gene flanked by the HIV-1 splice sites. These splice sites are not present within the gag mRNA expressed in the A3.9 cell line. Although the presence of a *cis*-acting repressor element (CRS) requires Rev *in trans *for expression of the p55 Gag protein [34].

The common feature of NOS-M4 and M4-M4 is the presence of two functionally similar basic domains. The dimer included two copies of the Rev basic domain while NOS-M4 contained one domain from Rev and one from Rex. The function of the HTLV-I Rex protein is similar to that of Rev and the basic domains from the two viral proteins bind to Importin β during nuclear import [35,36]. It is therefore likely that the two protein domains interact in a similar manner with other putative cellular cofactors. Previous reports have shown that Rex is able to rescue RRE containing RNA [32,33]. However, the binding site for Rex was shown to be different from the Rev binding site IIB [37]. Other studies did not confirm that Rex replaces Rev in *trans*-activity assays [38]. The co-transfection experiments in this study showed that Rex was not able to induce CAT expression from the Rev-dependent pDM138 reporters indicating that the Rex NOS domain did not bind the IIB or RRE sequences in this context (Figure 3C).

The rescue of activity of the mutants M4-M4 and NOS-M4 could be explained by at least two models. Both mutants comprise two basic domains with comparable functions. We found that Rex did not activate CAT expression from the Rev dependent pDM138 reporters. This indicated that the NOS-M4 mutant binds to the IIB RNA sequences by the Rev basic domain leaving the Rex domain free for other interactions. The previous observation that a peptide corresponding to the basic domain of Rev inhibited the *in vitro *splicing of RRE containing mRNAs underscores the functional importance of this region [39,40]. These results therefore support the suggestion that the basic domain participates in events other than binding to nuclear import factors and target RNA. This implies that at least one of the functional benefits of oligomer formation of Rev is that free basic domains can be exposed while the complex is tethered to RNA via other basic domains. Alternatively, the addition of extra sequences may have stabilized the structure of the otherwise unstable monomeric mutant, suggesting that dimer formation may be essential for obtaining a stable Rev structure.

## Conclusion

The present study showed that the activity of the negative monomeric M4 mutant was rescued by addition of an extra basic domain implying that two or more basic domains must be present within the complexes that bind to target RNA. This can be important for structural reasons or for leaving free basic domains for interaction with cellular co-factors when the Rev complex remains bound to viral RNA.

## Methods

### Plasmids

The plasmids pc*rev*-3xNLS and pc*rev*M4-3xNLS encoding Rev and the M4 mutant with three C-terminal copies of the SV40 large T antigen NLS were constructed by amplifying the *rev *coding region from pc*rev *and pc*rev*M4 using the primer pair catgccatggcaggaaga agcggag / ccgctcgagttctttagttcctgactccaa [1,29]. The PCR products were cloned into the *Nco*I-and *Xho*I-digested pCMV/myc/nuc vector (Invitrogen). The plasmid pc*rev*M4-M4 encodes an artificial M4 dimer with the monomers connected by a glycine rich linker. The *rev *coding region from pc*rev*M4 was amplified using the primer pairs tcgaagctagtcgacatctcctatg / cggggtaccgcctccttctttagctcc (PCR A) and cggggtaccggaatggcaggaagaagc / ctccagttggtagagagagcag (PCR B). The reverse primer in PCR B binds 70 nucleotides downstream from an *Xho*I site present in pc*rev*M4. The PCR product A was digested with *Sal*I and *Kpn*I while PCR B was digested with *Kpn*I and *Xho*I. The two PCR products were ligated together into the *Sal*I and *Xho*I digested pc*rev *vector. The NOS-M4 mutant contained the overlapping NLS/NOS from the HTLV-1 Rex protein fused to the N-terminal. The plasmid was constructed by inserting the *Nco*I-and *Xho*I digested fragment from pc*rev*M4-3xNLS into the *Nco*I-and *Xho*I digested pCMV/myc/cyto vector (Invitrogen), and inserting an *Nco*I flanked oligo encoding the overlapping NLS/NOS signal from the HTLV-1 Rex protein into the *Nco*I digested pc*rev*M4-myc vector. An overview of Rev and the Rev mutants is shown in figure 1A. The *rex *sequence was amplified from pcD-Srα Rex provided by Dr. Shida and cloned into the pc*rev *vector after excising the *rev *gene [41,42]. The Rev dependent pDM138-RRE and the Rex dependent pDM138-RxRE reporter plasmids contain the chloramphenicol acetyltransferase (CAT) gene and RRE or RxRE elements respectively within HIV intron sequences flanked by the 5' and 3' splice sites from the *env *gene [43,44]. In the plasmid pDM138-6xIIB the RRE sequence was replaced by six repeats of the IIB high affinity site for Rev binding allowing binding of six monomers [40]. The Rev dependent reporter plasmids are schematically shown in figure 1B.

### Transfections and cell lines

The A3.9 cell line stably expressing the gag mRNA fused to the RRE sequence was provided by M.L. Hammarskjöld and D. Rekosh. Transfection of A3.9 cells and COS-7 cells in 35 mm wells were carried out using Lipofectamine Reagent 2000 (GIBCO BRL Life Technologies) according to the manufacturer's recommendations. One coverslip was added to each 35 mm well and CAT and Rev expression by western blot and immunofluorescence respectively were analysed in parallel. Each 35 mm well was transfected with 1 μg of the pDM138 reporter plasmids. The amount of *rev *plasmid DNA varied from 0.2 – 2 μg in order to obtain the same amount of Rev or mutant protein expressed in the cells. The Rev protein levels were estimated by immunofluorescence. Of the control plasmids pc*rex *and pc*tat*, 4 μg and 1 μg respectively were added and the expression was confirmed by immunofluorescence.

### Immunofluorescence and western blot analysis

The cells were fixed or harvested 24 or 48 h post transfection for analysis by immunofluorescence and western blot respectively as previously described [28]. The immunofluorescence labeled cells were examined and the images were captured using the Leica DM RXA confocal scanning microscope with Leica PowerScan software attached. The figures were created using the program Adobe Photoshop version 3.0. The bands of the western blot analysis were scanned using an Agfa Snapscan 600 flatbed scanner and quantified using FUJIFILM LAS-1000 ProVer. 2.02 and Image Gauge V.3.45. The CAT bands were related to the activity of Rev set to 100 %, the negative control set to 0 % and the intensity of cellular bands representing the amount of cell lysate in the sample. Calculations were not performed when cellular background bands were not visible as in the experiments shown in figure 3.

#### Antibodies

The Rev proteins were detected by the anti-Rev Mab 8E7 [45].

Tat and Rex were detected using the Mabs 1D9 and 1F8 respectively (not shown) [42,46]. The anti-Gag p24 Mab for detection of full length Gag p55 expressed in A3.9 cells was supplied by H.C. Holmes, Medical Research Council, London, UK [47]. The polyclonal rabbit antibody for detection of the CAT protein was from Sigma.

## List of abbreviations used

RRE: Rev Responsive Element

HIV: Human Immunodeficiency Virus

CRS: *cis*-acting repressor sequences

Mab: Monoclonal antibody

CAT: Chloramphenicolacetyltransferase

NOS: Nucleolar localization signal

NLS: Nuclear localization signal

NES: Nuclear export signal

LMB: Leptomycin B

## Competing interests

The author(s) declare that they have no competing interests.

## Authors' contributions

CF: Vector design and construction. Transfections and immuoassays.

TA: Vector design and construction. Transfections and immuoassays.

PA: Vector design and construction.

JK: Experimental design, manuscript preparation

AMSz: Experimental design. Transfections and immuoassays. Manuscript preparation.

## References

[B1] Malim MH, Hauber J, Fenrick R, Cullen BR (1988). Immunodeficiency virus rev trans-activator modulates the expression of the viral regulatory genes. Nature.

[B2] Dayton AI, Terwilliger EF, Potz J, Kowalski M, Sodroski JG, Haseltine WA (1988). Cis-acting sequences responsive to the rev gene product of the human immunodeficiency virus. J Acquir Immune Defic Syndr.

[B3] Malim MH, Cullen BR (1991). HIV-1 structural gene expression requires the binding of multiple Rev monomers to the viral RRE: implications for HIV-1 latency. Cell.

[B4] Schwartz S, Felber BK, Pavlakis GN (1992). Distinct RNA sequences in the gag region of human immunodeficiency virus type 1 decrease RNA stability and inhibit expression in the absence of Rev protein. J Virol.

[B5] Cochrane AW, Jones KS, Beidas S, Dillon PJ, Skalka AM, Rosen CA (1991). Identification and characterization of intragenic sequences which repress human immunodeficiency virus structural gene expression. J Virol.

[B6] Maldarelli F, Martin MA, Strebel K (1991). Identification of posttranscriptionally active inhibitory sequences in human immunodeficiency virus type 1 RNA: novel level of gene regulation. J Virol.

[B7] Kalland KH, Szilvay AM, Brokstad KA, Saetrevik W, Haukenes G (1994). The human immunodeficiency virus type 1 Rev protein shuttles between the cytoplasm and nuclear compartments. Mol Cell Biol.

[B8] Meyer BE, Malim MH (1994). The HIV-1 Rev trans-activator shuttles between the nucleus and the cytoplasm. Genes Dev.

[B9] Cochrane AW, Perkins A, Rosen CA (1990). Identification of sequences important in the nucleolar localization of human immunodeficiency virus Rev: relevance of nucleolar localization to function. J Virol.

[B10] Malim MH, Hauber J, Le SY, Maizel JV, Cullen BR (1989). The HIV-1 rev trans-activator acts through a structured target sequence to activate nuclear export of unspliced viral mRNA. Nature.

[B11] Kjems J, Brown M, Chang DD, Sharp PA (1991). Structural analysis of the interaction between the human immunodeficiency virus Rev protein and the Rev response element. Proc Natl Acad Sci U S A.

[B12] Szilvay AM, Brokstad KA, Kopperud R, Haukenes G, Kalland KH (1995). Nuclear export of the human immunodeficiency virus type 1 nucleocytoplasmic shuttle protein Rev is mediated by its activation domain and is blocked by transdominant negative mutants. J Virol.

[B13] Fischer U, Huber J, Bolens WC, Mattaj IW, Luhrmann R (1995). The HIV-1 Rev Activation Domain is a Nuclear Export Signal that Accesses an Export Pathway used by Specific Cellular RNAs. Cell.

[B14] Wolff B, Cohen G, Hauber J, Meshcheryakova D, Rabeck C (1995). Nucleocytoplasmic transport of the Rev protein of human immunodeficiency virus type 1 is dependent on the activation domain of the protein. Exp Cell Res.

[B15] Powell DM, Zhang MJ, Konings DA, Wingfield PT, Stahl SJ, Dayton ET, Dayton AI (1995). Sequence specificity in the higher-order interaction of the Rev protein of HIV-1 with its target sequence, the RRE. J Acquir Immune Defic Syndr Hum Retrovirol.

[B16] Kjems J, Askjaer P (2000). Rev protein and its cellular partners. Adv Pharmacol.

[B17] Kjems J, Calnan BJ, Frankel AD, Sharp PA (1992). Specific binding of a basic peptide from HIV-1 Rev. Embo J.

[B18] Tiley LS, Malim MH, Tewary HK, Stockley PG, Cullen BR (1992). Identification of a high-affinity RNA-binding site for the human immunodeficiency virus type 1 Rev protein. Proc Natl Acad Sci U S A.

[B19] Cook KS, Fisk GJ, Hauber J, Usman N, Daly TJ, Rusche JR (1991). Characterization of HIV-1 REV protein: binding stoichiometry and minimal RNA substrate. Nucleic Acids Res.

[B20] Olsen HS, Cochrane AW, Dillon PJ, Nalin CM, Rosen CA (1990). Interaction of the human immunodeficiency virus type 1 rev protein with a structured region in env mRNA is dependent on multimer formation mediated through a basic stretch of amino acids. Genes Dev.

[B21] Brice PC, Kelley AC, Butler PJ (1999). Sensitive in vitro analysis of HIV-1 Rev multimerization. Nucleic Acids Res.

[B22] Zapp ML, Hope TJ, Parslow TG, Green MR (1991). Oligomerization and RNA binding domains of the type 1 human immunodeficiency virus Rev protein: a dual function for an arginine-rich binding motif. Proc Natl Acad Sci U S A.

[B23] Hope TJ, McDonald D, Huang XJ, Low J, Parslow TG (1990). Mutational analysis of the human immunodeficiency virus type 1 Rev transactivator: essential residues near the amino terminus. J Virol.

[B24] Madore SJ, Tiley LS, Malim MH, Cullen BR (1994). Sequence requirements for Rev multimerization in vivo. Virology.

[B25] Bogerd H, Greene WC (1993). Dominant Negative Mutants of Human T-Cell Leukemia Virus Type 1 Rex and Human Immunodeficiency Virus Type 1 Rev Fail to Multimerize In Vivo.. J Virol.

[B26] Szilvay AM, Brokstad KA, Boe SO, Haukenes G, Kalland KH (1997). Oligomerization of HIV-1 Rev mutants in the cytoplasm and during nuclear import. Virology.

[B27] Stauber RH, Afonina E, Gulnik S, Erickson J, Pavlakis GN (1998). Analysis of intracellular trafficking and interactions of cytoplasmic HIV-1 Rev mutants in living cells. Virology.

[B28] Gjerdrum C, Stranda A, Szilvay AM (2001). Functional role of the HIV-1 Rev exon 1 encoded region in complex formation and trans-dominant inhibition. FEBS Lett.

[B29] Malim MH, Bohnlein S, Hauber J, Cullen BR (1989). Functional dissection of the HIV-1 Rev trans-activator--derivation of a trans-dominant repressor of Rev function. Cell.

[B30] Thomas SL, Oft M, Jaksche H, Casar G, Heger P, Dobrovnik M, Bevec D, Hauber J (1998). Functional analysis of the human immunodeficiency virus type 1 Rev protein
oligomerization interface. J Virol.

[B31] Wolff B, Sanglier JJ, Wang Y (1997). Leptomycin B is an inhibitor of nuclear export: inhibition of nucleo-cytoplasmic translocation of the human immunodeficiency virus type 1 (HIV-1) Rev protein and Rev-dependent mRNA. Chem Biol.

[B32] Felber BK, Derse D, Athanassopoulos A, Campbell M, Pavlakis GN (1989). Cross-activation of the Rex proteins of HTLV-I and BLV and of the Rev protein of HIV-1 and nonreciprocal interactions with their RNA responsive elements. New Biol.

[B33] Hanly SM, Rimsky LT, Malim MH, Kim JH, Hauber J, Duc Dodon M, Le SY, Maizel JV, Cullen BR, Greene WC (1989). Comparative analysis of the HTLV-I Rex and HIV-1 Rev trans-regulatory proteins and their RNA response elements. Genes Dev.

[B34] Dundr M, Leno GH, Hammarskjold ML, Rekosh D, Helga-Maria C, Olson MO (1995). The roles of nucleolar structure and function in the subcellular location of the HIV-1 Rev protein. J Cell Sci.

[B35] Truant R, Cullen BR (1999). The arginine-rich domains present in human immunodeficiency virus type 1 Tat
and Rev function as direct importin beta-dependent nuclear localization
signals.. MCB.

[B36] Palmeri D, Malim MH (1999). Importin beta can mediate the nuclear import of an arginine-rich nuclear localization signal in the absence of importin alpha. Mol Cell Biol.

[B37] Solomin L, Felber BK, Pavlakis GN (1990). Different sites of interaction for Rev, Tev, and Rex proteins within the Rev-responsive element of human immunodeficiency virus type 1. J Virol.

[B38] Sakai H, Siomi H, Shida H, Shibata R, Kiyomasu T, Adachi A (1990). Functional comparison of transactivation by human retrovirus rev and rex genes. J Virol.

[B39] Kjems J, Frankel AD, Sharp PA (1991). Specific regulation of mRNA splicing in vitro by a peptide from HIV-1 Rev. Cell.

[B40] Kjems J, Sharp PA (1993). The basic domain of Rev from human immunodeficiency virus type 1 specifically blocks the entry of U4/U6.U5 small nuclear ribonucleoprotein in spliceosome assembly. J Virol.

[B41] Takebe Y, Seiki M, Fujisawa J, Hoy P, Yokota K, Arai K, Yoshida M, Arai N (1988). SR alpha promoter: an efficient and versatile mammalian cDNA expression system composed of the simian virus 40 early promoter and the R-U5 segment of human T-cell leukemia virus type 1 long terminal repeat. Mol Cell Biol.

[B42] Holden L (2000). A study of nuclear import of retroviral regulatory proteins using monoclonal antibodies and high efficiency eukaryotic expression vectors. Department of Molecular Biology.

[B43] Hope TJ, Bond BL, McDonald D, Klein NP, Parslow TG (1991). Effector domains of human immunodeficiency virus type 1 Rev and human T-cell leukemia virus type I Rex are functionally interchangeable and share an essential peptide motif. J Virol.

[B44] Hope TJ, Huang XJ, McDonald D, Parslow TG (1990). Steroid-receptor fusion of the human immunodeficiency virus type 1 Rev transactivator: mapping cryptic functions of the arginine-rich motif. Proc Natl Acad Sci U S A.

[B45] Kalland KH, Szilvay AM, Langhoff E, Haukenes G (1994). Subcellular distribution of human immunodeficiency virus type 1 Rev and colocalization of Rev with RNA splicing factors in a speckled pattern in the nucleoplasm. J Virol.

[B46] Valvatne H, Szilvay AM, Helland DE (1996). A monoclonal antibody defines a novel HIV type 1 Tat domain involved in trans-cellular trans-activation. AIDS Res Hum Retroviruses.

[B47] Ferns RB, Tedder RS, Weiss RA (1987). Characterization of monoclonal antibodies against the human immunodeficiency virus (HIV) gag products and their use in monitoring HIV isolate variation. J Gen Virol.

